# Fluid and burns in children: What we know and what we do not know—a retrospective analysis of the German Burn Registry from 2015 to 2022

**DOI:** 10.1007/s00431-024-05797-9

**Published:** 2024-10-22

**Authors:** Vasileios Vasileiadis, Safiullah Najem, Konrad Reinshagen, the German Burn Registry, Annette Aigner, Ingo Koenigs

**Affiliations:** 1 Altona Children’s Hospital, Department of Pediatric Surgery, Hamburg, Germany; 2 University Medical Center Hamburg-Eppendorf, Department of Pediatric Surgery, Hamburg, Germany; 3 Committee of the German Burn Registry, Berlin, Germany; 4 Universitätsmedizin Berlin, Corporate member of Freie Universität Berlin and Humboldt-Universität zu Berlin, Institute of Biometry and Clinical Epidemiology, Berlin, Germany; 5 Universitätsmedizin Berlin, Corporate member of Freie Universität Berlin and Humboldt-Universität zu Berlin, Center for Stroke Research, Berlin, Germany

**Keywords:** Major burns, Pediatric population, Fluid management, Parkland formula, Outcome

## Abstract

Fluid resuscitation is of great importance in the management of major burns. Various formulae have been described for calculating fluid management, especially in severely burned patients. Although the Parkland formula is widely used, its efficacy and clinical value are discussed controversially. We investigated the impact of deviation from calculated fluid volume by Parkland formula and the maintenance i.v. fluid (Parkland*) on the outcome of burned pediatric patients. Patients aged < 16 years with thermal injuries included in the German Burn Registry between January 2016 and December 2022 with a total body surface area ≥ 15% were analyzed. Using mixed-effect negative binomial regression, the association between a deviation from Parkland* in the administered fluid volume and the primary outcome length of hospital stay was estimated—additionally adjusted for known risk factors. As a secondary outcome, we use in-hospital mortality, evaluated descriptively. In 86.5% of patients, the administered fluid volume was lower than Parkland*, with pronounced deviation in the seven patients who died in the hospital. Descriptively and based on mixed-effect negative binomial regression models, we found that a positive deviation from Parkland* increases the number of hospital days, whereas a negative deviation may decrease them.

*Conclusion*: Very little is known about the role of administered resuscitation volumes for the outcome of pediatric severely burned patients. This study observed a tendency to a restricted resuscitation and its potential benefits in terms of length of hospital stay.

**Table:** 

**What is Known:** *• Fluid resuscitation is an important aspect of therapy in the acute phase of children with extensive burns.* *• The Parkland formula is routinely used formula for determining fluid requirements.*	
**What is New:** *• 86.5 % received less volume than determined by Parkland and a tendency to restricted resuscitation and its potential benefits in terms of length of stay was observed.* *• There is still considerable lack of clarity, and a strictly individualized protocol with the support of formulas is of crucial importance.*

**What is Known:**

*• Fluid resuscitation is an important aspect of therapy in the acute phase of children with extensive burns.*

*• The Parkland formula is routinely used formula for determining fluid requirements.*

**What is New:**

*• 86.5 % received less volume than determined by Parkland and a tendency to restricted resuscitation and its potential benefits in terms of length of stay was observed.*

*• There is still considerable lack of clarity, and a strictly individualized protocol with the support of formulas is of crucial importance.*

## Introduction  

Resuscitation is crucial in the management of severely burned patients and it is one of the most challenging tasks in the initial phase after burn injury. Major burns lead to an enormous inflammatory reaction causing microvascular hyperpermeability with massive fluid shifts into the interstitium resulting in edema and hypovolemia [1–4]. The correct amount of administrated fluid prevents complications and reduces mortality [5]. However, the definitions of “correct” vary greatly.

Over-resuscitation though can cause fluid extravasation and capillary leak with a higher risk of the development of acute respiratory distress syndrome, pneumonia, abdominal or limb compartment syndrome, cerebral edema, or multiple organ dysfunction [6–10]. Otherwise, delayed or inadequate fluid therapy results in organ under-perfusion and organ failure [3].

Various formulae exist for the calculating the infusion volume of severely burned patients [11]. Baxter introduced a formula in the late 1960s, later known as the Parkland formula, using 3.5 to 4 ml per of kg bodyweight per burned % of total body surface area (TBSA) as a guideline for resuscitation of severe burn patients [12]. In everyday practice and subsequent recommendations and guidelines, 4 ml is generally used, with few exceptions [13–15]. In the pediatric population, the maintenance volume requirement, as per 4–2-1 rule (4 ml/kg/h for the first 10 kg of weight, 2 ml/kg/h for the next 10 kg, and 1 ml/kg/h for each kilogram thereafter) or modified Holliday-Segar rule (100 ml/kg/day for the first 10 kg, 50 ml/kg/day for the second 10 kg, 20 ml/kg/day every kg thereafter) is added to the anticipated volume, i.e., the volume suggested by the Parkland formula [14, 16, 17]. Although the Parkland formula is the most widely used resuscitation protocol, its efficacy is increasingly questioned [18, 19].

The objective of this study was to retrospectively assess the volume of fluids administered in the first 24 h to paediatric patients with at least 15% TBSA burns. Furthermore, the study investigated the impact of calculated volumes by Parkland and its deviation on the outcome of the burned pediatric population in Germany, Switzerland, and Austria.

## Material and methods

### Study population

**Fig. 1:**
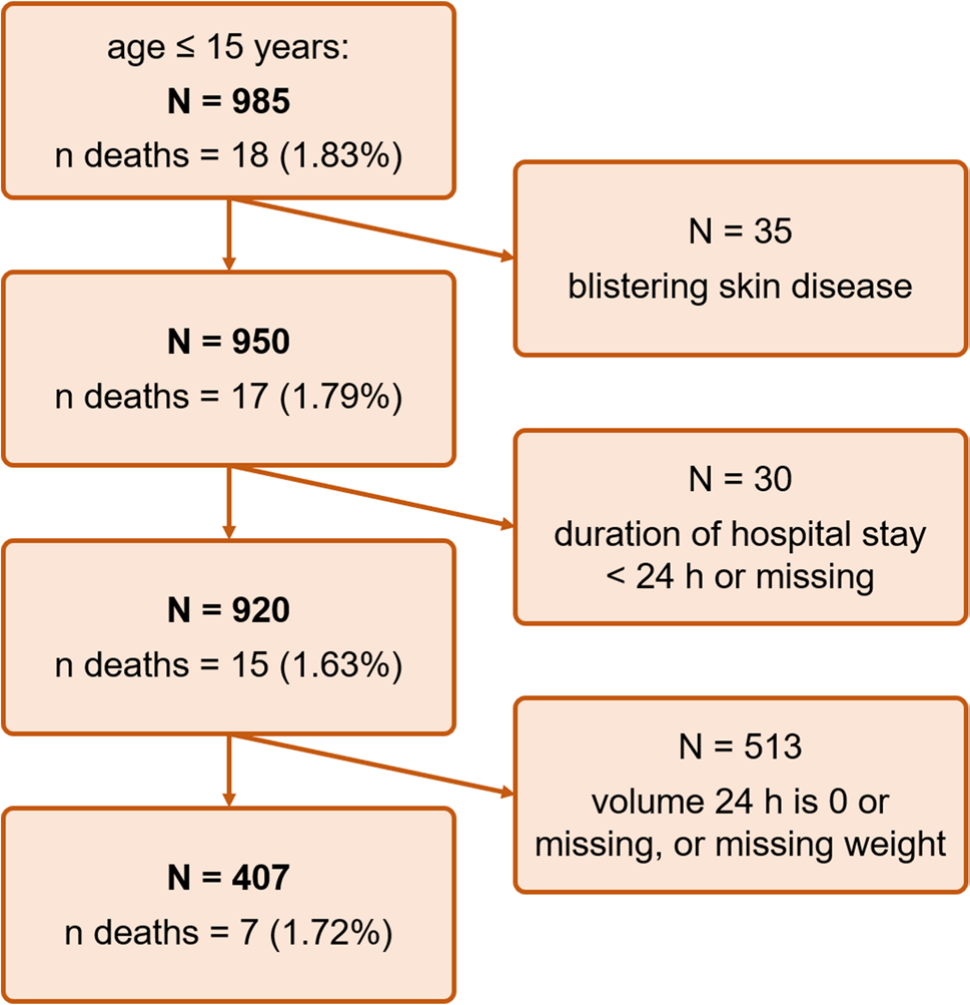
Study flowchart based on inclusion and exclusion criteria

The dataset is derived from the German Burn Registry (VR-DGV-project-ID 2020/01), collected between January 2016 and December 2022 [20]. This registry enrolls all children, irrespective of age, who require in-patient treatment due to thermal or comparable injuries such as acid or alkali injury or extensive blistering skin diseases, and all adults requiring intensive care treatment in Germany, Switzerland, and Austria. To date 57 hospitals and burn centers are incorporated in the registry, 43 pediatric hospitals, 17 adult hospitals, and 7 mixed hospitals, with annually varying numbers of burn patients. Burn centers and specialized clinics usually include their patients and are encouraged to do so by the guidelines. For this study, we excluded patients, who suffered blistering skin alterations, just as patients whose hospital stay was below 24 h, or this information was missing. Furthermore, also patients with missing weight or fluid uptake were excluded (Fig. 1).

### Calculation of fluid requirement

The total 24-h fluid requirement was defined as Parkland*, specified as the total daily fluid requirements for the burned child, individually calculated using Parkland’s formula to define “burns need” in addition to the maintenance i.v. fluid. The maintenance fluid was calculated according to the guidelines using the Holliday-Segar method (100 ml/kg/day for the first 10 kg, 50 ml/kg/day for the second 10 kg, 20 ml/kg/day every kg thereafter) [16, 21].

Therefore, the relative fluid volume deviation was defined as

$$\frac{administered\;volume\;within\;24h}{Parkland-based\;fluid+daily\;requirement}=\frac{administered\;volume\;within\;24h}{Parkland\ast},$$

where values above 1 indicate that more volume was administered than suggested by Parkland and the daily requirement together. Values below 1 indicate reduced volume administration. We assessed Parkland using 4 ml per kg bodyweight per % total body surface area, following leading clinical routine and guidelines. Therefore, Parkland* always corresponds to a calculation of 4 ml including basic requirements, unless otherwise stated. To account for the range mentioned in the original publication, we also conducted a sensitivity analysis using 3.5 ml.

### Statistical analysis

We report absolute and relative frequencies for categorical, medians along with interquartile ranges (IQR) for continuous variables, additionally with mean and standard deviation (SD) where meaningful—stratified by in-hospital mortality. The association between the deviation in fluid volume, and length of stay is graphically displayed using a locally weighted scatterplot smoothing (LOESS) estimate, the association with in-hospital mortality using boxplots.

The relative fluid deviation was then modelled such that positive and negative deviations could have different effects on the length of stay. In the unadjusted mixed-effect negative binomial regression model we therefore included the interaction between the relative extent of the deviation and the direction of the deviation. For this analysis, we excluded those individuals who experienced an in-hospital death. With a random intercept, we accounted for potential inherent differences between the individual centers. The results of this model are best interpretable with a graphical display linking the relative deviation to the length of stay. This model was then extended with known risk factors for a severe course of the disease—age, sex, whether it was a grade 3 burn or an inhalation trauma, and the routine of the treating center, the latter approximated with the number of patients given in the dataset. Based on these models, we derive unadjusted and adjusted rate ratios (RR), along with 95% confidence intervals (CI). As sensitivity analysis, we additionally performed a multiple imputation with 50 imputation datasets. Due to the very low number of in-hospital deaths recorded, we decided to evaluate associations between potential risk factors and this secondary outcome only descriptively.

Statistical analyses were performed with R, just as additional R packages [22–26].

The manuscript was peer-reviewed and approved by the Review Board of the Burn Registry of the German Society for Burn Medicine in accordance with the registry’s publication guidelines (VR-DGV-project-ID 2020/01).

## Results

Given the inclusion and exclusion criteria, the study population comprised 407 children, with a median age of 1 (IQR 1–6) and a mean age of 3.7 (SD 4.3), where the majority (62.4%) was male. Overall, 7.5% of children suffered from a sepsis, 7.2% from pneumonia, and 6.1% had an inhalation trauma. The median TBSA was 20 (IQR 16–25).

Scalds were the most common thermal injury (including contact with hot liquid, steam, or gas) and accounted for 74.2% (*n* = 302) of all patients included, followed by flame burns (17.0%) and high-voltage-caused injuries (2.7%). In terms of burn severity, 50.1% of all patients had a third-grade burn. The majority of patients died suffered a high-voltage accident.

Out of the 407 patients, 7 died during their hospital stay. Those who died were relevantly older (median = 9 vs 1, mean = 8.3 vs 3.7) and male (71.4% vs 62.2%), had more often a sepsis (60.0% vs 6.7%), pneumonia (80.0% vs 6.1%), an inhalation trauma (57.1% vs 5.2%), and the TBSA was higher (median = 82 vs 20). Based on the Parkland* formula their calculated fluid volume need was higher (median: 11540 vs 2287.80), just as their received fluid volume within 24 h (median: 4400 vs 1567) (Table 1).

**Table 1:** Patient characteristics stratified by outcome in-hospital mortality

	In-hospital mortality		Total (*n* = 407)
	No (*n* = 400)	Yes (*n* = 7)	
**Age**			
Median (IQR)	1.00 (1.0, 6.0)	9.0 (4.0, 13.0)	1.0 (1.0, 6.0)
Mean (SD)	3.7 (4.2)	8.3 (5.3)	3.7 (4.3)
**Age group** *n* (%)			
< 1	50 (12.5%)	0 (0.0%)	50 (12.3%)
1–4	235 (58.8%)	2 (28.6%)	237 (58.2%)
5–9	56 (14.0%)	2 (28.6%)	58 (14.3%)
10–15	59 (14.8%)	3 (42.9%)	62 (15.2%)
**Sex** *n* (%)			
Female	151 (37.8%)	2 (28.6%)	153 (37.6%)
Male	249 (62.2%)	5 (71.4%)	254 (62.4%)
**Height **(m)			
Median (IQR)	0.90 (0.8, 1.3)	1.51 (1.1, 1.7)	0.9 (0.8, 1.3)
Missing	62	0	62
**Weight** (kg)			
Median (IQR)	13.0 (11.0, 25.0)	30.0 (20.0, 58.5)	13.0 (11.0, 25.0)
**Body mass index**			
Median (IQR)	16.7 (15.2, 18.5)	20.4 (14.1, 21.7)	16.7 (15.1, 18.5)
Missing	62	0	62
**Sepsis**			
*n* (%)	22 (6.7%)	3 (60.0%)	25 (7.5%)
Missing	71	2	73
**Pneumonia**			
*n* (%)	20 (6.1%)	4 (80.0%)	24 (7.2%)
Missing	72	2	73
**Inhalation trauma**			
*n* (%)	21 (5.2%)	4 (57.1%)	25 (6.1%)
**Total body surface area** (%)			
Median (IQR)	20.0 (16.0, 25.0)	82.0 (53.0, 88.0)	20.0 (15.1, 18.5)
Mean (SD)	22.2 (9.7)	69.4 (24)	23.1 (11.8)
Parkland* (ml) = Parkland-based fluid + maintenance i.v. fluid			
Median (IQR)	2288 (1820, 3612)	11,540 (8405, 17,920)	2302 (1821, 3799)
**24-h volume** (ml)			
Median (IQR)	1567 (1000, 2600)	4400 (2371, 8698)	1575 (1000, 2660)
Absolute difference Parkland* & given volume in 24 h (ml)			
Median (IQR)	899 (431, 1529)	4740 (2674, 9150)	908 (452, 1548)
**Direction of deviation** *n* (%)			
Negative	346 (86.5%)	6 (85.7%)	352 (86.5%)
Positive	54 (13.5%)	1 (14.3%)	55 (13.5%)

*IQR* interquartile range, *SD* standard deviation

**Fig. 2:**
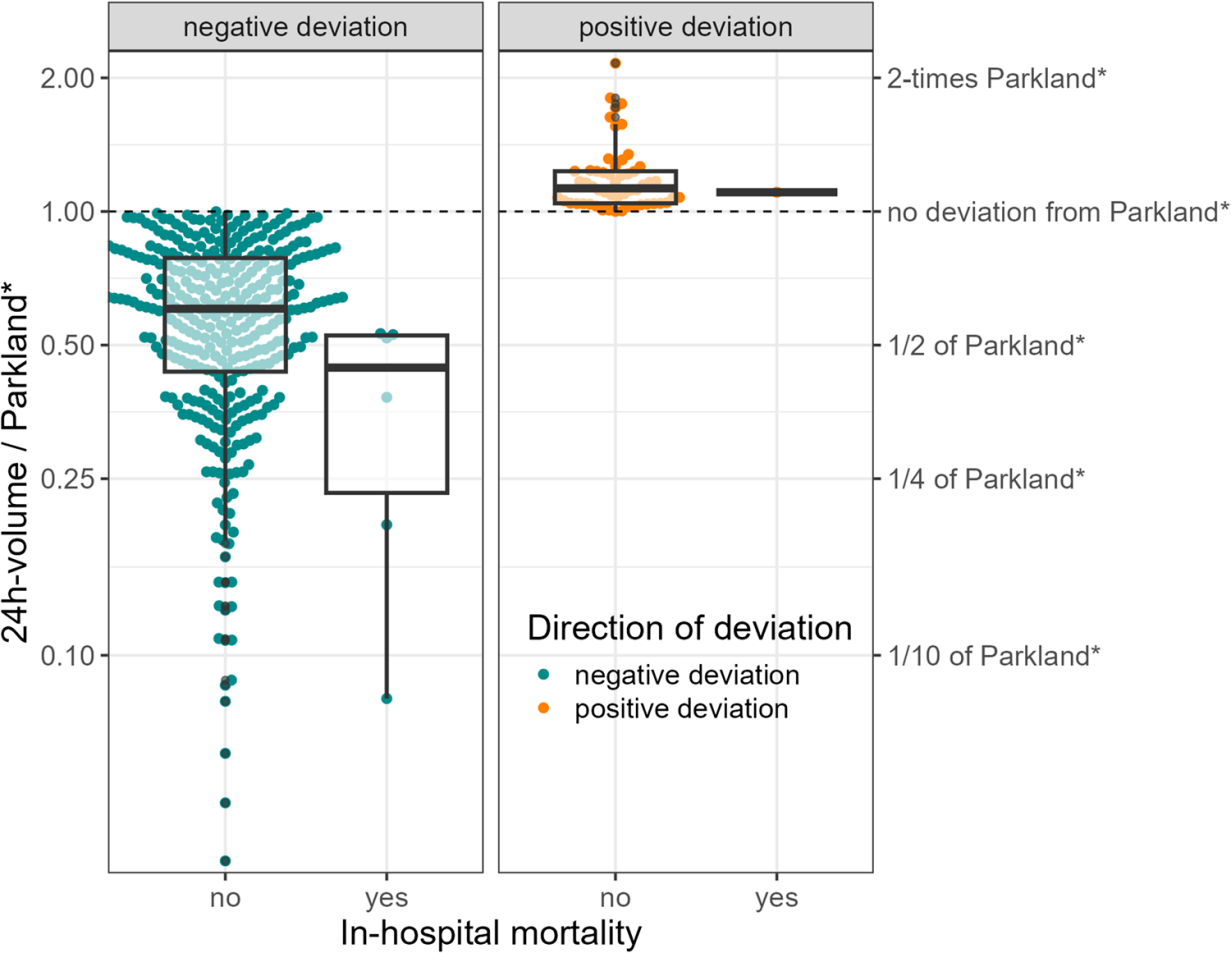
Relative deviation of the administered 24-h volume from the calculated fluid need (Parkland*), stratified by outcome in-hospital mortality and direction of deviation. *y*-axis, relative deviation of the administered 24-h volume from the calculated fluid need (Parkland*); *x*-axis, in-hospital mortality, colors indicate whether a positive or negative deviation is shown, regarding the in-hospital mortality

**Fig. 3:**
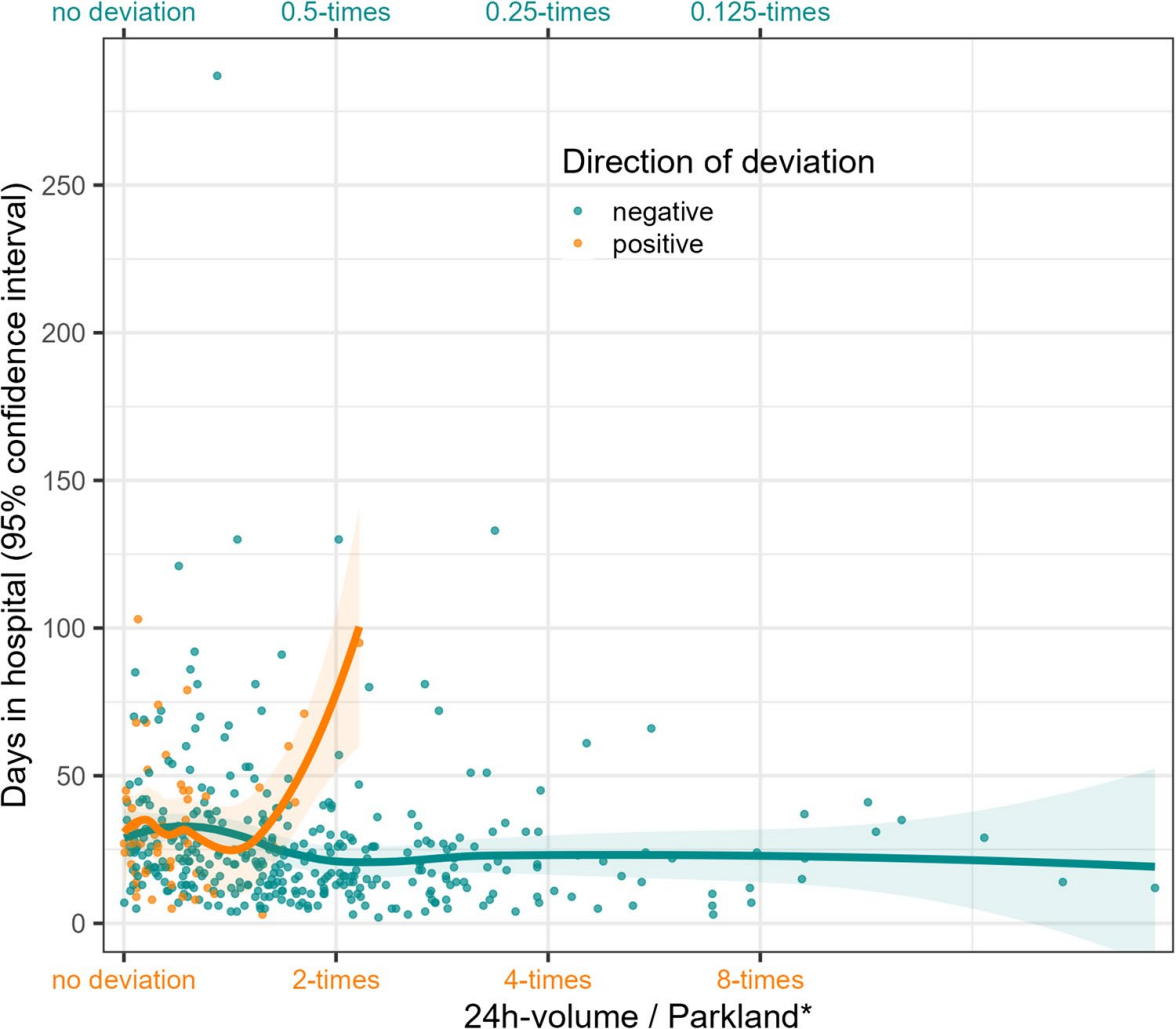
Scatterplot along with smoothing estimate (LOESS) for the association between length-of-stay and deviation from Parkland*. *y*-axis, days in hospital; *x*-axis, relative deviation of the administered 24-h volume from the calculated fluid need (Parkland*), colors indicate whether a positive or a negative relative deviation is shown, range of LOESS estimates determined by the range of observations

Overall, some deviation from Parkland* was always observed, where a negative deviation was more frequent (86.5%), i.e., less fluid was administered. The absolute deviation from Parkland* was higher for those who died (median = 4740 vs 899), just as the relative deviation, in both directions (Table 1 and Fig. 2). The association between the deviation from Parkland* and the length of stay is descriptively not as clear—positive deviations from Parkland*, i.e., giving more fluid, are associated with an increase in hospital days, negative deviations from Parkland* do not relate to the days in hospital or potentially decrease them (Fig. 3).

**Fig. 4:**
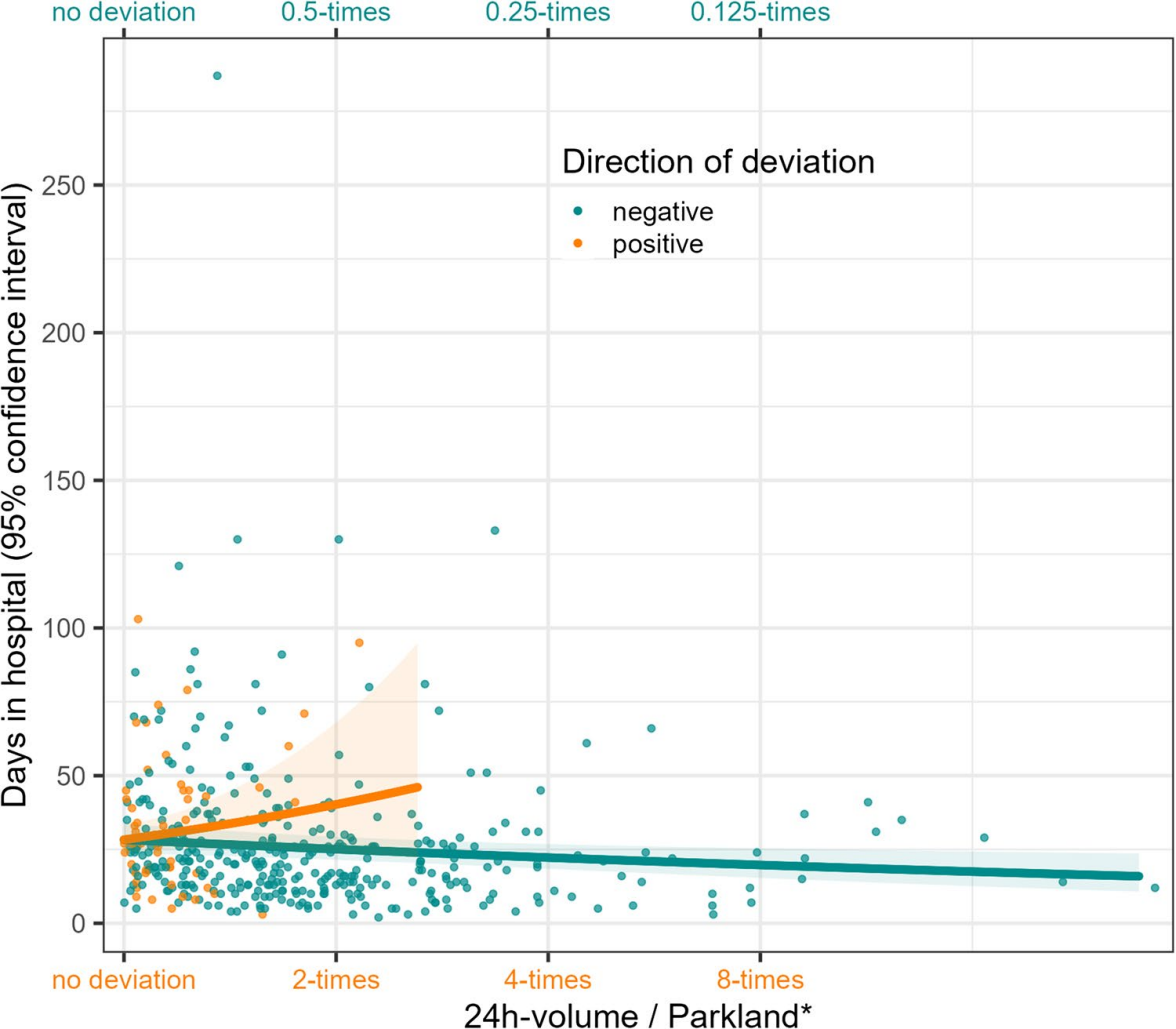
Scatterplot along with predicted association between the relative deviation from Parkland* and the length-of-stay. *y*-axis, days in hospital; *x*-axis, relative deviation of the administered 24-h volume from the calculated fluid need (Parkland*), colors indicate whether a positive or a negative relative deviation is shown, predictions derived from a mixed-effect negative binomial model without further adjustment, range of predicted values determined by the range of observations

First, we modelled the association between the relative deviation from Parkland and the length of stay without controlling for other variables. Based on this negative binomial regression we derived predictions to plot the estimated associations (Fig. 4). We found that a positive deviation from Parkland* increases the number of hospital days, more specifically giving twice as much as Parkland* suggests, increases the rate of hospital days 1.42-fold (RR = 1.42, 95% CI 0.83–2.33). Differently, a negative deviation from Parkland* decreases the hospital days, where giving only half of what Parkland* suggests reduces the rate of hospital days 0.89-fold (95% CI 0.81–0.97). Given multiple imputation, the results still indicate that a positive deviation is associated with a higher rate of days spent in hospital, but the estimated effects are smaller with increased imprecision in the estimation (RR for positive deviation: 1.20, 95% CI 0.24–5.98; for negative deviation: 0.89, 95% CI 0.68–1.16). Overall, these results indicate that there might be an overestimation in the effect of a positive deviation from Parkland*, but especially this result can only be estimated with high imprecision in any case.

**Fig. 5:**
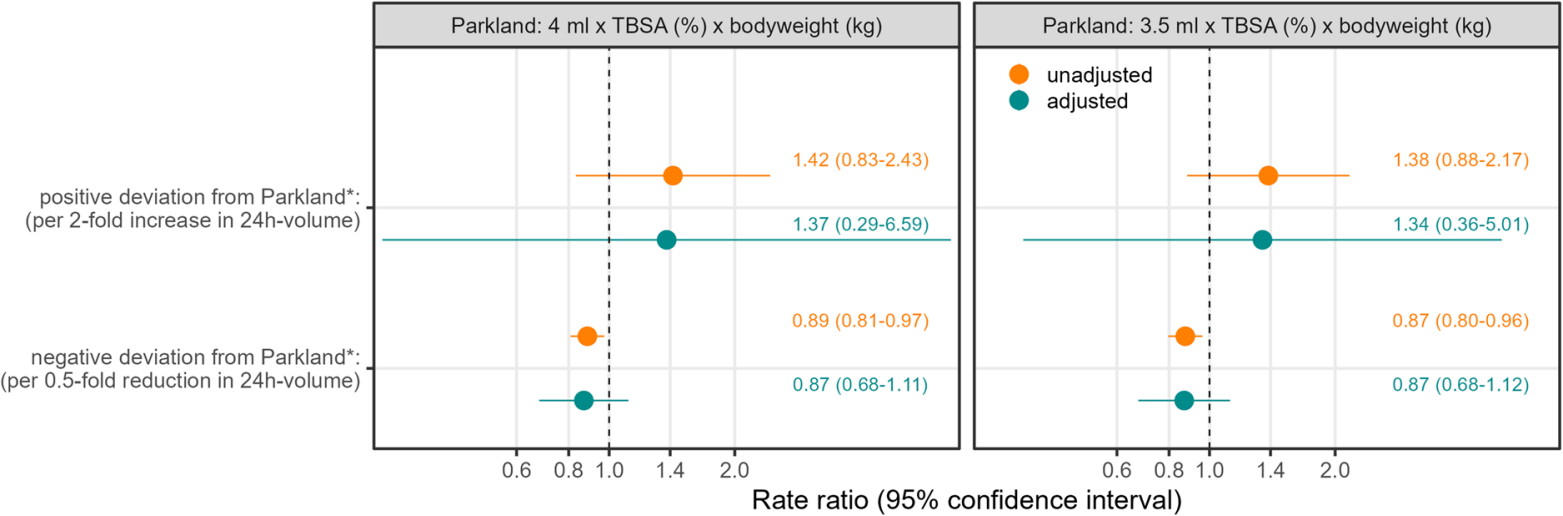
Rate ratios for the association between the deviation from Parkland* (4 ml and 3.5 ml) and the length-of-stay, unadjusted and adjusted for known risk factors. Rate ratios are displayed along with 95% confidence intervals (CI), rate ratios are derived from mixed-effect negative binomial regression for outcome length of hospital stay, colors indicate whether the rate ratios stem from a model where all other risk factors are included or not; known risk factors were defined as age, sex, grade 3 burn, inhalation trauma, and routine of the treating center

The associations of both a negative and positive deviation remain largely the same when all other variables are introduced to the model, showing that the effect of deviation from Parkland* is independent of age, sex, whether it was a grade 3 burn or an inhalation trauma, and the routine of the treating center (Fig. 5). Using instead the “bottom edge” of the Parkland formula of 3.5 ml per kg bodyweight % TBSA, the individual effect estimates are somewhat attenuated, i.e., using 3.5 ml the effect sizes are somewhat smaller than using 4 ml. Regardless of the chosen fluid amount, there is a lower number of observed positive deviations from Parkland, which is why this effect estimate is marginally more affected.

## Discussion

Based on retrospectively collected registry data of pediatric burn patients over a 7-year period, we aimed to assess the Parkland formula. More precisely, the association between the relative deviation of administered fluid volume within the first 24 h after trauma and the two outcomes, length of hospital stay and in-hospital mortality, were evaluated, where the reference for deviation was the fluid need, estimated by the Parkland formula plus the daily requirements, derived by Holliday-Segar method (Parkland*). Epidemiological data such as cause of accident, age, gender, and other patient characteristics are similar to other publications from Europe, Canada, and US, such that a representative character of our cohort can be assumed [27–31].

We found that in 86.5% of patients the fluid volume was lower than Parkland*. The vast majority of children are significantly below the Parkland formula in childhood; this applies to the entire cohort, but also to the small group of deceased. The deviation was more pronounced in those patients who died in the hospital. However, the pleasingly low mortality prevents further meaningful investigations due to the small number of cases. Descriptively and based on a mixed-effect negative binomial regression model we found that a positive deviation from Parkland* increases the number of hospital days, whereas a negative deviation decreases them.

The analyses were conducted using 4 ml/kg bodyweight/% TBSA, as this is the most commonly used value from the original publication by Baxter et al. Additional analyses using the lower limit of the Parkland formula (3.5 ml) were also performed but did not reveal relevant differences. The effect on the length of stay of a positive deviation was slightly reduced in size, further indicating that lower infusion volumes lead to better outcomes than higher volumes, which underscores the benefit of a more conservative infusion approach.

The mainly negative deviation reflects the new German interdisciplinary guidelines for pediatric burn treatment in burns ≥ 15% TBSA; besides the basic need, an additional burn requirement of 3–4 ml/kg/% TBSA should be administered, especially in children who do not show adequate drinking behavior due to injury and its subsequent treatment (sedation, etc.). These guidelines also suggest a close monitoring of circulatory parameters and urine output, and fluid intake must be controlled individually for each child or adolescent in the sense of early goal therapy [32].

Our study showed discrepancies in the resuscitation protocols used among the various burn centers, supporting the results of Pisano et al. [33] analyzing different pediatric burn centers in the US. Parkland is the favorite resuscitation formula in an international study among 70% of burn specialists worldwide [18]. Various studies showed an excessive over-resuscitation in the majority of the patients of up to 100% [34–36]. However, in our study, the majority of the patients received less than Parkland calculated. Daniels et al. [37] and Arlati et al. [38] demonstrated lower mortality rates in adults after restricted resuscitation. In our study six of the seven patients who died were under-resuscitated with respect to the Parkland* formula. Contrarily, but based on more patients, we found a relation between the deviation from Parkland* and the length of hospital stay, where giving less fluid decreases the length of stay. This relates to the study of Walker et al. [6], who described a significant association between length of stay and fluid volume administration in children. Furthermore, this study identified significant differences in the length of stay between over- and adequately resuscitated patients, a result which is in line with the findings of Blumeti et al. in the adult population [19]. Due to the lack of further outcome criteria such as patients/family satisfaction or quality of life, only the length of stay allows an assessment of the cause and the result.

The positive association between inhalation trauma and length of stay relates to the findings by Smith et al. in non-pediatric population [39]. The present study only showed a very weak or even negligible direct effect of patients’ sex on the length of hospital stay, given the adjustment for other variables. This confirms current literature [35, 40], which also shows no gender differences in the outcome, contrary to older publications, which analogous to the still widely used Abbreviated Burn Severity Index, describe a poorer prognosis in females [41]. This relates well to the modified abbreviated burn severity index score published by Bartels et al. [42]. Depth of injury had an expected influence on clinical course, the rate of in-hospital days was two-fold in third-degree burns.

The Parkland resuscitation fluid volume is based on TBSA, which is subjective, often inaccurate, and mostly overestimated, which is resulting in failure of resuscitation, usually in infusion volume that is too high [43, 44]. The estimates of body surface area vary greatly in studies with pronounced effects on fluid volume. Parvizi et al. showed that in two example patients (2 and 4 years) the body surface area estimated by registrars, burn specialists, and specialist nurses differed from 2.5 to 25%, respectively 8.5 to 40%. This led to a difference in the calculated fluid volume between the smallest and the greatest estimation of 1080 ml and respectively 2016 ml [38, 45]. For this reason, a correct determination of the body surface area is crucial.

Due to the many potential sources of error, the initially estimated body surface area must be promptly re-evaluated and fluid management must be closely monitored and quickly adapted if necessary. Advanced hemodynamic monitoring with pulse contour analysis and transpulmonary thermodilution is considered the most effective tool for managing infusion therapy in adults [42, 46]. However, its use in children is very rare, primarily due to the device’s size [37]. For this reason, urine output remains the most relevant endpoint in routine pediatric clinical practice. Although urine output is not an ideal parameter even in childhood, it is a “better” parameter than in adults because of the general absence of kidney disease or underlying conditions that can affect kidney function. A target criterion in guidelines is a urine output of 1–2 ml/kg/h in infants and young children, 0.5–1 ml/kg/h in older children [32]. It was shown that the amount of infusion administered in adulthood was significantly lower than the Parkland calculation when urine output is an immediate control mechanism [36]. However, adaptation should take place just as quickly when reducing the amount as when increasing it. This does not seem to be the case in clinical practice, as too little urine output per kg bodyweight per hour usually leads to a rapid increase of the infusion volume, but too much urine does not lead to a correspondingly rapid reduction of the infusion [47]. In childhood, a dynamic and continuously assessed, highly individualized fluid replacement strategy is the ideal approach. Whenever possible and necessary, extended invasive haemodynamic monitoring should serve as the basis for volume control and intensive therapy, just as it does in adult patients [42, 46].

## Strengths and limitations

The present study is based on a large international registry of burn patients, using data of over 400 pediatric patients across 30 burn centers in three countries. We were able to evaluate the Parkland formula both with respect to length of hospital stay, just as in-hospital mortality.

However, the number of the patients who died was extremely low, and even more reduced due to the exclusion criteria and missing documentation. It is also possible that some thermal injury cases are not included in the registry database. The major limitation of this study is the lack of information on patient monitoring through arterial pressure and urine output [48]. Although the latter has been controversially discussed by Boehm et al. and Peeters et al. [32, 49], it can be important for monitoring a pediatric population supported by hemodynamic parameters and burn resuscitation formulas. Additionally, the specific formula used to calculate the resuscitation volume in the included centers is unknown. No data on oral feeding and calculation method of daily fluid requirements are provided. Consequently, these two parameters have now been added to the German Burn Registry protocol. Furthermore, the better physical condition and prognosis of patients with shorter in-hospital stays could lead to reduced fluid resuscitation and thereby causing a negative deviation from the Parkland formula.

## Conclusion

Ultimately, it must be acknowledged that we still know relatively little about the optimal resuscitation for children with thermal injuries. Overall, the impression is that “less is sometimes more/better.”

Prospective multinational randomized studies would be necessary to investigate the optimal fluid volume, but their practical feasibility is questionable due to ethical concerns and small sample sizes in pediatric populations.

The Parkland formula is helpful in the immediate phase following trauma, but requires rapid adaptation based on urine output and invasive circulatory monitoring in severely injured patients.

A tendency to a more restricted resuscitation, with potential benefits in terms of length of hospital stay was observed and has been incorporated into the German interdisciplinary guidelines for pediatric burn treatment, recommending lower resuscitation volumes [21].

The question remains whether a new formula is needed for calculating fluid requirements in burn-injured children or whether a purely urine-output-controlled infusion management with constant adjustments represents a more clinically individualized and sophisticated approach.

## Data Availability

Full data are available on request from authors.

## Abbreviations

CI: Confidence interval
IQR: Interquartile range
SD: Standard deviation
Parkland*: Parkland-based fluid + maintenance i.v. fluid
RR: Rate ratio
TBSA: Total body surface area
